# Patient’s age rather than severity of the arrhythmia influences the cost of medical treatment of atrioventricular nodal or atrioventricular reciprocating tachycardia

**DOI:** 10.1007/s10840-016-0167-9

**Published:** 2016-08-03

**Authors:** Michal M. Farkowski, Mariusz Pytkowski, Aleksander Maciag, Dominik Golicki, Ilona Kowalik, Marcin Czech, Piotr Rucinski, Hanna Szwed

**Affiliations:** 1 The 2nd Department of Coronary Artery Disease, Institute of Cardiology, Spartanska 1 St., 02-637 Warsaw, Poland; 2 Department of Experimental and Clinical Pharmacology, Medical University of Warsaw, Warsaw, Poland; 3 Business School, Warsaw University of Technology, Warsaw, Poland; 4 Arion Hospitals Group, Lublin, Poland

**Keywords:** AVNRT, AVRT, Cost, Medical treatment

## Abstract

**Purpose:**

Radiofrequency ablation (RFA) is considered the treatment of choice in cases of atrioventricular nodal reciprocating tachycardia (AVNRT) and atrioventricular reciprocating tachycardia (AVRT). Published studies suggest a considerable time gap between the onset of the arrhythmia, correct diagnosis, and RFA which may reach 10–15 years. The cost of medical treatment during that period may be substantial. The aim of the study was to calculate the annual direct medical cost of medical treatment of patients with AVNRT and AVRT and identify potential factors influencing this cost.

**Methods:**

Based on the consumption of particular resources and the unit costs of services in 2013, we calculated the annual direct medical cost of care for patients with AVNRT and AVRT in Poland. We adopted the public payer’s and societal perspectives. Data on health resources was collected with a structured questionnaire and medical records of patients scheduled for RFA. Additional analyses were performed to identify factors influencing this cost.

**Results:**

We enrolled 82 patients: mean age 43.9 ± 14.1 years old and mean symptom duration before the RFA 13.0 ± 11.3 years. The median annual cost of medical treatment was 546 USD [312–957], 411 € [278–786], and 616 USD [369–1044], 464 € [235–721], for the public payer and the common perspective, respectively, with hospitalizations being the main cost component. In multivariate analysis, only the age of the patient significantly influenced this cost.

**Conclusions:**

The annual cost of medical treatment of AVNRT or AVRT is substantial and dependent on the age of the patient rather than the severity of the arrhythmia (NCT01594814).

## Introduction

Atrioventricular nodal reciprocating tachycardia (AVNRT) and atrioventricular reciprocating tachycardia (AVRT) are two of supraventricular tachycardias (SVTs) which are benign in nature in most cases but result in a significant impairment of health-related quality of life [1–3]. In both cases, radiofrequency ablation (RFA) is considered a curative procedure with high effectiveness and low rate of serious complications [4–7]. While atrial fibrillation remains the main target of RFA in many countries, SVTs remain a significant portion of RFA procedures in Europe [8]. The relatively non-specific clinical presentation and frequent problems to obtain the electrocardiographical (ECG) documentation of those arrhythmias lead to a long gap between the onset of symptoms and RFA which in many cases may exceed 15 years [5, 9, 10]. Costs of medical treatment of SVT: repetitive consultations, hospitalizations, and diagnostic tests, due to recurrent episodes of arrhythmias before the RFA may be substantial [1].

The aim of this study was to calculate the annual direct medical cost of medical treatment of AVNRT and AVRT and identify the factors influencing this cost.

## Methods

This was a pre-specified subanalysis of the Patients’ Perspective on Radiofrequency Catheter Ablation of AVRT and AVNRT (PPRA) Study (NCT01594814) [9]. PPRA was a single-center, prospective, cohort study performed in a tertiary-care cardiological center. We enrolled patients admitted for the RFA of AVNRT and AVRT. The inclusion criteria were written informed consent, over 18 years of age, and sufficient knowledge of the Polish language to independently complete the questionnaires. The main exclusion criterion was any serious comorbidity significantly impairing the quality of life. During the hospitalization, eligible patients were asked to complete questionnaires concerning general socio-economic status and the quality of life (Patient Perception of Arrhythmia Questionnaire, PPAQ and EQ-5D-3L) and a questionnaire concerning health care resource utilization during the year preceding the RFA [11–13].

Data on the following resources was collected: outpatient specialist consultations, outpatient additional tests (echocardiography, 24-h Holter ECG, ECG stress testing, blood laboratory tests), acute care—emergency department visits and sanitary transport, hospitalizations, drugs—name, and dosage. Public insurance and private consultations and tests were collected separately.

### Country-specific information

Poland is a Central European country with 36 million inhabitants and is divided into 15 voivodships, local government units roughly equal to a county. The Polish National Health Fund (NHF) is the single, nationwide public insurer which covers the cost of hospitalizations, acute care, and outpatient health care in Poland. The NHF is divided into 15 departments, one for every voivodship. Departments are financially independent from one another and have similar tariffs for hospitalization (via diagnosis-related groups, DRG) and specialist outpatient consultations but may differ in lump sums for acute care providers. In terms of hospitalizations and acute care, there are no significant private insurers but the outpatient specialist consultations are easily commercially available outside the NHS and the out-of-pocket costs for the patient are reasonable. The primary care is organized as a network of general practitioners who are recompensed by the NHF on the basis of a lump price for every signed patient regardless of the number of visits, type of illness, or treatment. Similarly, emergency departments and sanitary transport providers are paid as a lump sum based on a projected annual number of consultations/transports rather than actual fee-for-service.

### Analysis

We calculated direct medical costs of care adapting the public payer and the common perspective of the public payer and patient. The public payer perspective covered all costs borne solely by the NHF. The common perspective covered all costs borne by both the NHF and all of patients’ out-of-pocket cost related to the arrhythmia treatment. A time horizon was restricted to 1 year, so no discounting was applied. Costs of care were calculated in Polish Zloty and converted to US dollars and Euro according to the Polish National Bank mean rate of exchange for the year 2013. Median cost of care over patients with AVNRT or AVRT was calculated as a median cost of care of individual patients. Calculations for all costs covered by the Polish NHS via DRGs or cardiological specialist outpatient care were based on unit costs derived from the NHF official documents and resource utilization declared by patients. The cost for in- and outpatient services was determined as the mean cost for such services for three voivodships in 2013. For outpatient visits, we adopted a conservative assumption of maximizing the cost for the NHF based on additional tests performed by the consultant. We also assumed all hospitalization costs were calculated based on the DRG for patients with heart arrhythmia without serious complications (DRG E62 group). The mean cost of emergency department consultation and sanitary transport was calculated as a median cost derived from five hospitals from three voivodships and seven providers of sanitary transport from three voivodships. To further increase the accuracy of the analysis, additional information on the mean cost of emergency department consultation and sanitary transport was calculated based on official data obtained from the Mazovian NHF. Costs of reimbursed drugs were calculated based on a mean yearly dose and the mean cost of 1 mg of the drug derived from the Ministry of Health’s (MoH) official sales prices. All mentioned NHF and MoH documents were valid for the 30th of December 2013.

Costs of specialist consultations and additional tests performed in the private sector (patients’ out-of-pocket costs) were calculated based on resource utilization declared by patients and data derived from the official websites of two of the biggest country-wide providers. Costs of non-reimbursed drugs (patients’ out-of-pocket costs) were calculated based on a mean yearly dose and the mean cost of 1 mg of the drug derived from the website of a country-wide provider.

The severity of the index arrhythmia was estimated based on the patients’ quality of life measured using the widely recognized, generic EQ-5D-3L questionnaire [12]. The EQ-5D-3L comprises five dimensions (mobility, self-care, usual activities, pain/discomfort, anxiety/depression) with three levels (problems, some problems, extreme problems) within each dimension and a visual analog scale (VAS). The responses for all five dimensions can be converted into a single summary index by applying a formula that essentially attaches values (also called weights) to each of the levels in each dimension. The summary index equal to “1” means no problems at all while −0.55 means extreme problems in all five dimensions and the worst measurable health state. In our previous analysis, the EQ-5D-3L accurately described the quality of life of patients with different numbers and lengths of arrhythmia episodes and different accompanying signs and symptoms and arrhythmia’s influence on everyday life [9]. To take into account all those factors and simplify the present analysis, we decided to use the summary index of EQ-5D-3L as a single measure of the “severity” of the arrhythmia.

The study protocol was approved by the local Institutional Review Board and was in full compliance with the Declaration of Helsinki.

### Statistical analysis

The Kolmogorov-Smirnov test was used to check for normal distribution of continuous data. Normally distributed continuous data were presented in terms of mean ± standard deviation*. Non-normally distributed continuous data were expressed in terms of percentiles (costs: median and inter-quartile range) * or median, min, and max (number of procedures) and were compared across two groups by non-parametrical Mann-Whitney *U* test and across three groups by non-parametrical ANOVA Kruskal-Wallis test. Categorical variables were summarized in terms of frequencies and percentages. Multivariable linear regression analysis was applied to verify the independent association of cost of care in AVNRT and AVRT with predictor yields in univariate analysis. First, we constructed a linear regression model in which the cost of care was entered as an independent variable (because of asymmetry >2.0, it was log-transformed) and all potential explanatory variables from the univariate analysis were included in the model. After, a backward selection procedure as used with the significance set at *p* < 0.05 to remove a covariate from the model. To avoid assumptions about linearity, residuals were examined. All hypotheses were two-tailed with a 0.05 type I error. All analyses were conducted using SAS software (version 9.2., SAS Institute Inc., NC, USA).

## Results

Between January 2012 and August 2013, among 96 consecutive potentially eligible patients, 82 were enrolled. The reasons for exclusion were lack of informed consent (12 patients) and insufficient knowledge of the Polish language (2 patients). The most important baseline characteristics of the patients included in the analysis are presented in Table 1. Delta wave in the surface ECG was present in 25.6 % of patients, and 13.4 % had a history of atrial fibrillation. There were no cases of clinically significant heart failure or coronary artery disease, and 20.7 % of patients had hypertension.

**Table 1:** Baseline characteristics of enrolled patients

	*N* = 82
Age (years ± SD)	43.9 ± 14.1
Female gender	49 (59.8 %)
Symptom duration before ablation (years ± SD, range [IQR])	13.0 ± 11.3; 10 [3–20]
AVNRT	45 (55 %)
AVRT	37 (45 %)
History of concomitant atrial arrhythmia	16 (19.5 %)
EQ-5D-3L	
Utility	0.87 [0.80–1.00]
VAS	71.0 ± 18.0
Dwelling place	
Countryside or town <25,000 inhabitants	44 (54.3 %)
Town ≥25,000 inhabitants	37 (45.7 %)
Professional activity	
Employed	55 (67.1 %)
Unemployed	11 (13.4 %)
Retirement/disability pension	10 (12.2 %)
Other	6 (7.3 %)

*AVNRT* atrioventricular nodal reciprocating tachycardia, *AVRT* atrioventricular reciprocating tachycardia, *SD* standard deviation, *IQR* inter-quartile range, *VAS*, visual analog scale

At the time of hospitalization, 26.2 % of patients were on beta-blocker, 1.2 % on propafenone, and none on amiodarone or sotalol. The median utility measured by EQ-5D-3L was decreased—0.87 [0.80–1.00] as expected before the RFA of the index arrhythmia.

The mean/median medical care resource utilization due to arrhythmia during 1 year preceding the RFA is presented in Table 2.

**Table 2:** Resource utilization due to arrhythmia during 1 year in the public and private sectors

	Number of patients (%)	Mean ± SD	Median [range]
Public sector			
Cardiologist cons.	59 (71.9 %)	3.5 ± 3.2	3 [1–15]
ECHO	55 (67.1 %)	1.9 ± 1.8	1 [1–12]
ECG Holter	42 (51.2 %)	1.9 ± 1.3	1 [1–6]
Thyroid tests	26 (31.7 %)	1.4 ± 0.8	1 [1–4]
ECG stress test	11 (13.4 %)	1.1 ± 0.3	1 [1–2]
ED visit	61 (74.4 %)	2.7 ± 3.2	2 [1–24]
Hospitalization	49 (59.8 %)	1.4 ± 0.9	1 [1–6]
Sanitary transport	34 (41.5 %)	2.3 ± 2.3	2 [1–12]
Private sector			
Cardiologist cons.	41 (50.0 %)	2.4 ± 1.5	2 [1–6]
ECHO	24 (29.3 %)	1.7 ± 1.6	1 [1–8]
ECG Holter	19 (23.2 %)	1.3 ± 0.8	1 [1–4]
Thyroid tests	9 (1.0 %)	1.6 ± 1.1	1 [1–4]
ECG stress test	1 (1.2 %)	1	1

*SD* standard deviation, *ECHO* echocardiography, *ECG* electrocardiography, *ED* emergency department

### Cost of care

The calculated annual median cost of care over patients with AVNRT or AVRT is presented in Table 3. The single highest component cost of care was the cost of hospitalization—312 USD [0–312], 235 € [0–235], followed by costs of emergency department visits—144 USD [72–288], 108 € [54–217]. Costs of drugs were relatively low and borne entirely by the patient—14 USD [0–40], 11 € [0–30].

**Table 3:** Annual cost of care of patients with AVNRT and AVRT using general and Mazovian NHF cost data

	Median cost [IQR]
General costs	
Public payer perspective	546 USD [312–957], 411 € [235–721]
Common perspective	616 USD [369–1044], 464 € [278–786]
Mazovian NHF costs	
Public payer perspective	463 USD [234–791], 349 € [176–596]
Common perspective	548 USD [345–871], 413 € [260–656]

*AVNRT* atrioventricular nodal reciprocating tachycardia, *AVRT* atrioventricular reciprocating tachycardia, *NHF* National Health Fund, *USD* United States dollar

The cost of AVNRT calculated from the common perspective was slightly higher than that of AVRT: 579 USD [358–801], 496 € [318–971], vs. 459 USD [423–1289], 436 € [270–603], *p* = 0.04; public payer costs were similar between AVNRT and AVRT.

The cost of care significantly increased with age (Table 4). The cost of care of patients living in the countryside or towns <25,000 inhabitants were significantly higher than that of patients living in bigger towns or cities: 619 USD [361–1210], 466 € [272–912], vs. 463 USD [220–647], 349 € [166–487] (*p* = 0.01), and 659 USD [422–1354], 496 € [318–1020], vs. 539 USD [297–760], 406 € [223–572] (*p* = 0.01), for the payer and the common perspective, respectively. Also, higher education of the patient slightly increased the public payers’ cost but not the cost calculated from the common perspective.

**Table 4:** Relationship between patients’ age (in years) and cost of care in AVNRT and AVRT

	Age <33	Age 33–51	Age >51	*p*
Public payer perspective	370 USD [96–609] 279 € [72–459]	515 USD [345–745] 388 € [260–561]	922 USD [418–1744] 694 € [314–1313]	*p* = 0.0002
Common perspective	495 USD [213–683] 373 € [160–514]	605 USD [423–820] 456 € [318–618]	943 USD [543–1849] 710 € [409–1393]	*p* = 0.0004

*AVNRT* atrioventricular nodal reciprocating tachycardia, *AVRT* atrioventricular reciprocating tachycardia, *NHF* National Health Fund, *USD* United States dollar

The multivariate analysis of factors influencing the median cost of care in the univariate analysis is presented in Table 5: only age remained a significant factor although patients with diabetes and hypertension and patients on beta-blocker and sotalol tended to be older. The severity of the arrhythmia expressed as a generic quality of life was not a significant factor in the univariate analysis and hence was not included in the multivariable model.

**Table 5:** Baseline model without selection variables. Model contains all significant variables from the univariate analyses. Dependent value is log(NHF costs)

	Regression coefficient ± SEM	*p*
Age [in decades]	0.22 ± 0.10	0.024
Dwelling place	−0.12 ± 0.24	0.627
Education	−0.35 ± 0.26	0.181
Coronary artery disease	−0.02 ± 0.39	0.960
Hypertension	0.14 ± 0.32	0.662
Sotalol	0.08 ± 0.36	0.826
Beta-blocker	0.05 ± 0.34	0.883
Atrial fibrillation	0.44 ± 0.36	0.220

*NHF* National Health Fund

The sensitivity analysis of arrhythmia cost calculation was performed by calculating all results using lower and higher boundaries of confidence intervals of the respective component costs—the sensitivity analysis confirmed the results of the primary analysis.

## Discussion

To our knowledge, this is the first study to calculate annual costs of medical treatment of patients with AVNRT or AVRT. While the RFA is the cornerstone of therapy for such patients, a lengthy time between the onset of symptoms and RFA could generate significant costs for both the health care system and the patient alike. In our analysis, the annual cost of medical treatment was 546 USD, 411 €, and 616 USD, 464 €, for the public payer and the common perspective, respectively, and the main component of this cost was hospitalization due to arrhythmia. The cost of DRG for the catheter ablation of SVT in Poland was about 8048 USD, 4335 €, in 2013. Median costs were lower when we used data from the Mazovian NHF. As mentioned before, in Poland, emergency departments and sanitary transport providers are paid as a lump sum based on a projected number of consultations/transports rather than actual fee-for-service. In this case, consultations/transports over the projected amount are not paid for at all by the NHF and hence the lower average cost of single consultation/transport and lower median cost of care. Patients treated medically in the USA generated over 6000 USD of costs of care during 5 years due to 3.7 ± 1.3 clinic visits, 17 emergency department visits, and 2 hospitalizations [1]. In our analysis, at least half of the patients were hospitalized due to arrhythmia and the median number of emergency department visits was 2 during 1 year. The easiest explanation for the high number of hospitalization in our study is again NHF: a patient who is hospitalized is recompensed to the hospital by a means of the DRG group based on the indication and treatment received while in the emergency department only as a lump sum regardless of the clinical situation. Under these conditions, the hospitals are interested in admissions rather than only the acute treatment because they get both the compensation for DRG and emergency department lump sum.

A high proportion of the median cost of care in our analysis was due to acute situations: hospitalizations, emergency department visits, and sanitary transport. Those costs are hard to avoid without successful and permanent treatment of the index arrhythmia. Medical treatment is generally considered an accessory to the RFA which is also apparent in our study: only about 20 % had an antiarrhythmic drug prescribed while scheduled for RFA. As expected, median yearly cost of drugs was low and borne entirely by the patient—about 11 €.

One of the most interesting findings of our study was that a very important factor influencing the cost of care was the age of the patient (Table 4). One probable explanation of this finding is the higher proportion of additional tests to exclude concomitant diseases especially coronary artery disease (CAD) in older patients since chest discomfort and dyspnea accompanying arrhythmia may mimic CAD. In our previous analysis, men were more likely to undergo echocardiography and stress testing mainly to exclude significant CAD [9]. This is important since older patients with AVNRT are not uncommon [14]. Other possible explanation is that our study group was too small to highlight the importance of higher prevalence of comorbidities in this group and their independent influence. Other obvious factors such as symptom duration before the RFA and arrhythmia severity measured as a quality of life (EQ-5D-3L) did not affect the cost of medical treatment of AVNRT or AVRT.

The annual cost of medical treatment of AVNRT or AVRT was significantly lower than the average annual cost of heart failure or CAD treatment in Poland: 1787 € and 2254 €, respectively [15, 16]. In both cases, the cost depended heavily on the severity of the disease measured as a New York Heart Association or Canadian Cardiovascular Society classification and hospitalizations being the main cost component. Surprisingly, in our analysis, severity of the arrhythmia was not associated with cost of care but hospitalizations remained the main cost component. An estimated cost of atrial fibrillation treatment, 1010 €, in the Polish setting was again higher than that in AVNRT or AVRT with hospitalizations among the main drivers of costs [17].

### Limitations

This study has its limitations, the most important being the medium sample size and its single-center character, both limiting the generalizability of study findings. We tried to limit the potential selection bias by enrolling a wide range of patients with different socio-economic status and severities of the arrhythmias. All calculations were performed based on an assumption of maximizing health care providers’ income; hence, presented costs for the public payer might have been slightly overestimated. On the other hand, we did not incur costs of the primary care which in Poland are borne as a lump sum regardless of the disease and treatment so were not considered “arrhythmia-dependent.”

The data on utilized health resources was gathered up to 1 year prior to the RFA to minimize the potential recall bias. During the pilot phase of PPRA, it appeared that patients had significant problems with recalling information on outpatient visits or acute treatment over longer periods than about 1 year. By omitting data from longer periods of time, we limited the generalizability and it is hard to reliably estimate the potential influence of this kind of data on the results of the study.

## Conclusions

The annual cost of medical treatment of AVNRT or AVRT is substantial and dependent mainly on the age of the patient. Bearing in mind study limitations and issues concerning international generalizability of economic analyses, this study suggests that early diagnosis and definitive treatment of AVNRT or AVRT in younger patients may lower costs of treatment of those arrhythmias and alleviate the economic burden on local health care payers.
